# A Rare Cause of Abdominal Pain in Childhood: Cardiac Angiosarcoma

**DOI:** 10.21470/1678-9741-2017-0095

**Published:** 2018

**Authors:** Elvan Caglar Citak, Murat Ozeren, M. Kerem Karaca, Derya Karpuz, Feryal Karahan, Eda Bengi Yilmaz, Yuksel Balci, Pelin Ozcan Kara, Rabia Bozdogan Arpaci

**Affiliations:** 1 Department of Pediatric Oncology, Mersin University Faculty of Medicine, Mersin, Turkey.; 2 Department of Cardiovascular Surgery, Mersin University Faculty of Medicine, Mersin, Turkey.; 3 Department of Radiation Oncology, Mersin University Faculty of Medicine, Mersin, Turkey.; 4 Department of Radiology, Mersin University Faculty of Medicine, Mersin, Turkey.; 5 Department of Nuclear Medicine, Mersin University Faculty of Medicine, Mersin, Turkey.; 6 Department of Pathology, Mersin University Faculty of Medicine, Mersin, Turkey.

**Keywords:** Hemangiosarcoma, Heart, Heart neoplasms, Child

## Abstract

Cardiac angiosarcomas are extremely rare in childhood, they are rapidly progressive tumours that often present themselves as diagnostic dilemmas, resulting in delayed diagnosis. Also, extracardiac manifestations, including abdominal pain, are extremely rare in patients with intracardiac tumors. We herein present the case of a 15-year-old girl who presented with abdominal pain. Echocardiography and thoracic computed tomography showed right atrial mass. The patient underwent surgery, chemotherapy, and radiotherapy. Eight months after treatment, abdominal recurrence was detected. The abdominal mass was resected, and radiotherapy and new chemotherapy protocol were given. The present case illustrates a rare case of primary cardiac angiosarcoma posing a diagnostic dilemma in an adolescent girl.

**Table:** 

Abbreviations, acronyms & symbols	
ACT	= Activated coagulation time
CD31	= Cluster of differentiation 31
CT	= Computed tomography
ERG	= Erythroblast transformation specific regulated gene-1
FDG	= (18)F-fluorodeoxyglucose
FLI-1	= Friend leukemia integration-1
HHV-8	= Human herpesvirus 8
HMB45	= Human melanoma black 45
IMA	= Ifosfamide/mesna/adriamycin
PET	= Positron emission tomography
SMA	= Smooth muscle actin

## INTRODUCTION

Primary cardiac tumors are extremely rare in children, with an incidence varying between 0.0017 and 0.28%^[1]^. The majority of them are benign and sarcomas represent the most common malignant tumors^[1]^. Among primary adult cardiac sarcomas, angiosarcoma is more common than rhabdomyosarcoma, but it remains an unusual occurrence. In this report, we present a 15-year-old girl who was admitted to the emergency department with a complaint of severe abdominal pain and who was diagnosed with cardiac angiosarcoma.

## CASE REPORT

**Fig. 1:**
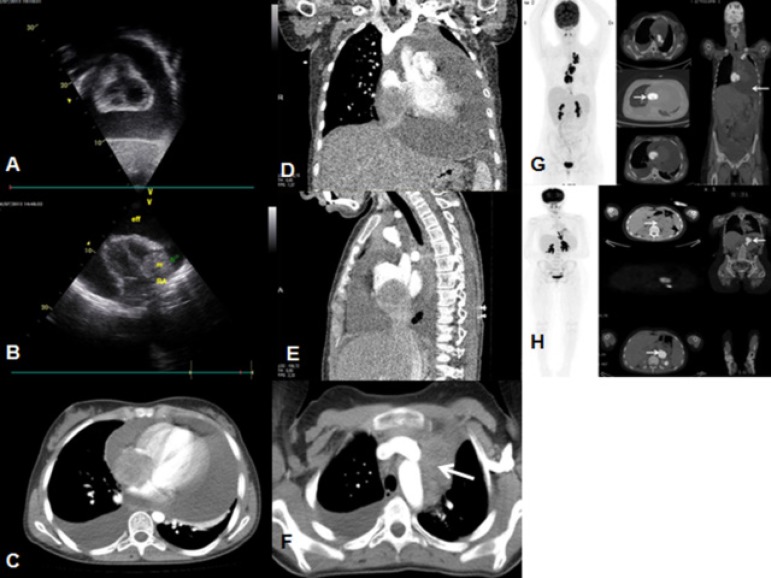
A) Pericardial effusion in transthoracic echocardiography. B) Hyperechogenic mass arising from the right atrium and invading the right ventricle on transthoracic echocardiography in four-chamber view. C) Axial thoracic computed tomography (CT). D) Coronal thoracic CT. E) Sagittal thoracic CT; soft tissue lesion (5 cm in size) filling the right atrial lumen and invading the right ventricle and, partially, the suprahepatic segment; pericardial and pleural effusion. F) Soft tissue lesion in left upper mediastinum (white arrow). G) (18)F-fluorodeoxyglucose (FDG) positron emission tomography/computed tomography (PET/CT) images demonstrate hypermetabolic mass (SUVmax: 14.29) in the right atrium, bilateral pleural and pericardial effusion, and mediastinal metastatic lymphadenopathy (SUVmax: 15.07). H) FDG PET/CT images demonstrate hypermetabolic mass (SUVmax: 15) in the medial left kidney.

A 15-year-old, Syrian refugee girl who was a high school student was admitted to the emergency department with a history of 15 days of abdominal pain. On physical examination, she is 160 cm tall and weighs 50 kg. She had no history of cancer in her family. She presented with abdominal tenderness, hepatosplenomegaly and muffled heart sounds. Abdominal and thoracic computed tomography (CT) showed hepatosplenomegaly and solid mass in the right atrium and in the left mediastinum. There was a massive pericardial and moderate right pleural effusion (Figure 1). Echocardiogram showed massive pericardial effusion and a solid tumor in the right atrium expanding towards the right ventricule (Figure 1). (18)F-fluorodeoxyglucose (FDG) positron emission tomography/computed tomography (PET/CT) scan revealed increased FDG uptake in the mediastinum and over the heart. Pleural and pericardial effusion and intra-abdominal free fluid were also seen (Figure 1).

### Surgical Technique

**Fig. 2:**
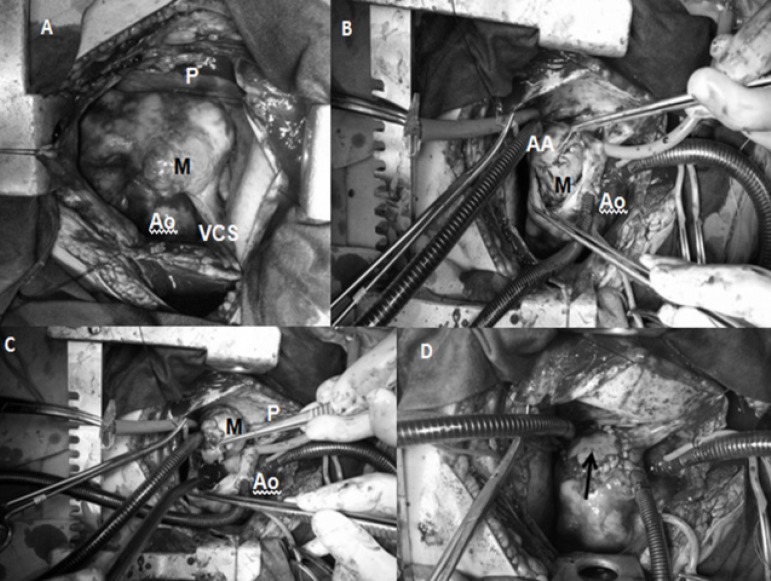
A) Mass (M) before right atriotomy. B) Mass infiltrating atrial appendage (AA). C) Removal of the mass. D) The defect was repaired by pericardial tissue. Ao=aorta; P=pericardium; VCS=vena cava superior

The patient was taken to the operation room under semi-urgent conditions. Tense pericardium due to excessive pericardial liquid was observed after midline sternotomy. The pericardium was opened and 1500 cc of serous liquid were evacuated from the cavity. Macerated epicardial appearance and probable malignant infiltration areas inside the right ventricle wall were seen. Patient was heparinized (3 mg/kg) and the activated coagulation time (ACT) obtained was above 400 seconds. Total cardiopulmonary bypass was started via aortic and bicaval cannulation. Cardioplegia was infused after crossclamping of the ascending aorta. The right atrium was opened with vertical incision. A solid atrial mass that was completely invading the right atrial wall and also prolapsing through the tricuspid valve and causing partial obstruction of right ventricle inflow was observed. The mass was totally excised along with the right atrial wall. This resected area was repaired by using glutaraldehyde-treated autologous pericardium (0.1%) (Figure 2). After completion of repair procedure, de-airing was done. Patient was weaned off the cardiopulmonary bypass without any inotropic support. The postoperative period was uneventful.

### Postoperative Course

**Fig. 3:**
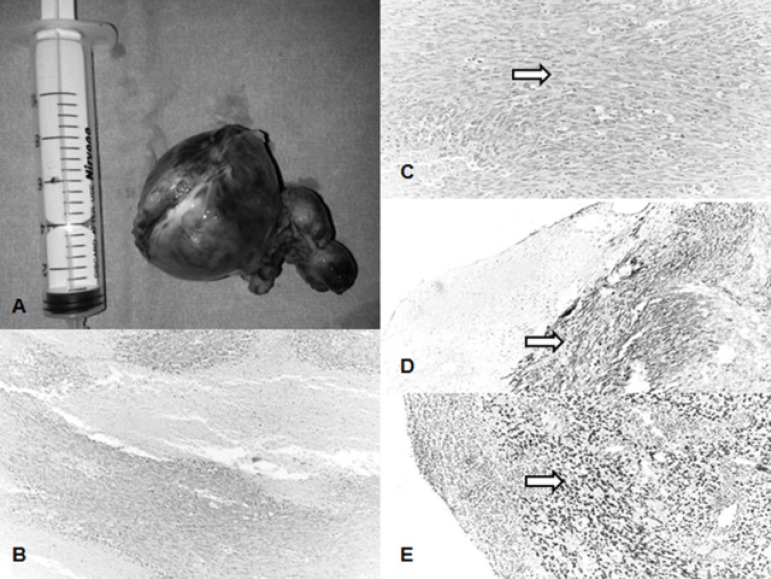
A) Gross pathological picture of the tumor. B) Cellularity and necrosis of the angiosarcoma (HE, x100). C) Angiosarcoma cells round, oval, or spindle shaped (arrow) (HE, x200). D) Tumor cells (arrow) were immunohistochemistry cytoplasmic stained by cluster of differentiation 34 (CD34, x200). E) Tumor cells (arrow) show nuclear positive immunohistochemical staining with friend leukemia integration-1 (FLI-1) (CD34, x100).

The mass in the right atrium was reported as an angiosarcoma. On immunohistochemical analysis, tumor cells were positive for cluster of differentiation 31 (CD31), CD34, friend leukemia integration-1 (FLI-1) and erythroblast transformation specific regulated gene-1 (ERG), but they were negative for keratin, desmin, smooth muscle actin (SMA), myogenin, Human melanoma black 45 (HMB45) and human herpesvirus 8 (HHV-8) (Figure 3). Ifosfamide/mesna/adriamycin (IMA) chemotherapy combination was administered. After 6 cycles of chemotherapy, the patient was in complete remission. A total dose of 50.4 Gy was administered to the operating margin and mediastinal lymph nodes using 3-D conformal radiotherapy. Eight months after the therapy was finished, the patient was admitted with abdominal pain. Abdominopelvic and thoracic CT revealed an irregularly contoured mass in the medial region of the right kidney. PET/ CT scan showed a new lesion, and it was excised and reported as an angiosarcoma. The patient received 3 cycles of gemcitabine/ docetaxel chemotherapy combination. After chemotherapy, a dose of 45 Gy was administered to the operating margin. Since the follow-up period of 20 months from the date of the diagnosis, the patient is in remission for 6 months from the last recurrence.

## DISCUSSION

Primary angiosarcoma of the heart is an exceptional diagnosis in pediatric population. Cardiac tumors, either benign or malignant, are difficult to diagnose due to their rarity, variety and nonspecificity of the symptoms.

Primary cardiac angiosarcomas in all age groups mostly occur in the right atrium^[2]^. The most common complaints presented are dyspnea and chest pain, usually related to a malignant cardiac effusion^[2]^. The pericardium is frequently involved with a right sided angiosarcoma; cardiac tamponade and pericardial effusion are common complications^[2,3]^. Our patient had a massive pericardial effusion and malignant cells were seen in cytologic examination. Extracardiac manifestations, including abdominal pain, are extremely rare in patients with intracardiac tumors, like our case.

The rarity of this diagnosis has made it difficult to standardize therapy, but surgical treatment is the main option. A multimodality treatment including preoperative and/or postoperative chemotherapy and/or radiotherapy seems beneficial for overall or progression-free survival. Using modern radiation techniques, radiotherapy on the heart seems to be safe, without major cardiac toxicity^[3]^.

To our knowledge, only 9 children cases of primary cardiac angiosarcoma were detailed reported, including ours^[4,5]^. The average age at presentation is 12.3 years. There were 4 girls and 4 boys; one patient's gender was not determined. Eight patients had right atrium involvement. Most patients presented with dyspnea or chest pain. Our patient had an extracardiac manifestation. Six of 9 patients had a metastatic disease at the time of diagnosis. Six patients died with a progressive disease.

A study about the frequency of cancer among Syrian and Turkish children has revealed that most of cancer types were similar in frequency, but the percentage of children with neuroblastoma and bone tumours was significantly higher in refugees^[6]^. Preliminary outcomes in Syrian refugee children with cancer were similar to the outcomes in Turkish children^[6]^.

As a result, children with abdominal pain have to be examined for extra-abdominal diseases and cancer.

**Table:** 

Authors' roles & responsibilities	
ECC	Conception and study design; manuscript redaction or critical review of its content; final manuscript approval
MO	Conception and study design; realization of operations; manuscript redaction or critical review of its content; final manuscript approval
MKK	Conception and study design; realization of operations; manuscript redaction or critical review of its content; final manuscript approval
DK	Conception and study design; realization of echocardiographic investigation; manuscript redaction or critical review of its content; final manuscript approval
FK	Conception and study design; manuscript redaction or critical review of its content; final manuscript approval
EBY	Conception and study design; realization of radiotherapy; manuscript redaction or critical review of its content; final manuscript approval
YB	Conception and study design; realization of radiological evaluations; manuscript redaction or critical review of its content; final manuscript approval
POK	Conception and study design; realization of PET CT evaluations; manuscript redaction or critical review of its content; final manuscript approval
RBA	Conception and study design; realization of pathological evaluations; manuscript redaction or critical review of its content; final manuscript approval
